# Virulence of the Lyme disease spirochete before and after the tick bloodmeal: a quantitative assessment

**DOI:** 10.1186/s13071-016-1380-1

**Published:** 2016-03-07

**Authors:** Irene N. Kasumba, Aaron Bestor, Kit Tilly, Patricia A. Rosa

**Affiliations:** Laboratory of Zoonotic Pathogens, Rocky Mountain Laboratories, National Institute of Allergy and Infectious Diseases, National Institutes of Health, Hamilton, MT 59840 USA; Current address: Center for Vaccine Development, Department of Geographic Medicine, University of Maryland School of Medicine, Baltimore, MD 21201 USA

**Keywords:** *Borrelia burgdorferi*, *Ixodes* ticks, Conditional priming, Lyme disease spirochete

## Abstract

**Background:**

*Borrelia burgdorferi*, the tick-transmitted agent of Lyme disease, adapts to different environments as it cycles between an arthropod vector and vertebrate host. Signals encountered during nymphal tick feeding prior to transmission activate a regulon that is controlled by the alternative sigma factors RpoN and RpoS, which are required for mammalian infection. The ingested bloodmeal also provides nutrients that stimulate spirochete replication. Although the influence of tick feeding on spirochete growth and gene expression is well documented, a quantitative assessment of spirochete virulence before and after tick feeding has not been made.

**Methods:**

Homogenates were prepared from unfed and fed infected *Ixodes scapularis* nymphs that had acquired *B. burgdorferi* as larvae. Serially diluted tick homogenates were needle-inoculated into mice to determine the infectious dose of tick-derived spirochetes before and after the bloodmeal. Mouse infection was assessed by sero-reactivity with *B. burgdorferi* whole cell lysates on immunoblots and attempted isolation of spirochetes from mouse tissues. Viable spirochetes in tick-derived inocula were quantified by colony formation in solid media.

**Results:**

We found that an inoculum containing as many as 10^4^*B. burgdorferi* from unfed ticks is largely non-infectious, while the calculated ID_50_ for spirochetes from fed ticks is ~30 organisms. Engineered constitutive production of the essential virulence factor OspC by spirochetes within unfed ticks did not confer an infectious phenotype.

**Conclusion:**

Conditional priming of *B. burgdorferi* during tick feeding induces changes in addition to OspC that are required for infection of the mammalian host.

## Background

*Borrelia burgdorferi*, the spirochetal agent of Lyme borreliosis, is maintained in a natural infectious cycle involving small rodents and *Ixodes* ticks [1–4]. As *B. burgdorferi* cycles between vector and host, it senses external cues and adapts by making gene products appropriate for each environment (reviewed in [5, 6]). This ability to detect sudden changes in external stimuli and modulate gene expression is mediated by a relatively small set of known regulatory proteins and sigma factors [7]. Sigma 70 (σ^70^, RpoD) directs the expression of most *B. burgdorferi* genes, including some that are tightly regulated at various stages of the infectious cycle. Two alternative sigma factors,σ^54^ (RpoN) and σ^s^ (RpoS), function in concert to direct the expression of a smaller set of spirochete genes required primarily in the mammalian host [8–12].

The RpoN-RpoS regulon is modulated as *B. burgdorferi* traverses between the mammalian host and tick vector. When larvae feed on an infected mammal and acquire spirochetes, the RpoN-RpoS cascade is de-activated; it is re-activated when molted nymphs take a blood meal and spirochetes in the tick midgut sense accompanying stimuli, such as an influx of nutrients and changes in temperature and pH (reviewed in [5]). One hallmark of the RpoS-dependent changes that occur during tick feeding is induction of OspC [8, 13]. OspC is an outer surface lipoprotein that is required by *B. burgdorferi* to initiate infection of the mammalian host, but subsequently down regulated during persistent infection [14, 15]. Because of the direct link between activation of the RpoN-RpoS regulon and synthesis of factors required for tick transmission [16] and host infection [8, 14, 15, 17–19], it is anticipated that spirochetes residing in distinct environments within the tick vector (unfed versus fed) will also display significant differences in mammalian infectivity.

An early study by Piesman and colleagues found that unfed nymphal tick homogenates were non-infectious when inoculated into mice and concluded that unfed ticks contained too few spirochetes to constitute an infectious dose [20]. Although these and more recent studies clearly document the influence of stimuli within the starved and fed tick vector on spirochete physiology [20–33], the impact of these factors on spirochete infectivity in the mammalian host has not been quantitatively assessed.

In this study, we directly compared the infectious dose in mice by needle inoculation of defined numbers of viable spirochetes derived from ticks before and after blood feeding. We use the term “attenuated” to refer to spirochetes that are genetically identical to wild-type organisms but exhibit reduced infectivity because of their biological state. We will refer to the event that elicits infectivity in attenuated spirochetes as “conditional priming”. Our results demonstrate that viable *B. burgdorferi* in unfed ticks are highly attenuated in their ability to infect mice, even with an inoculum of >7×10^3^ organisms engineered to constitutively produce the virulence factor OspC. We conclude that conditional priming of *B. burgdorferi* during tick feeding induces critical changes, in addition to OspC production, that specifically prepare the spirochete for infection of the mammalian host.

## Methods

### *Borrelia burgdorferi* strains and culture conditions

Infectious clone B31-A3, derived from the B31 type strain of *B. burgdorferi* (ATCC 35210), was used as the wild type (WT) strain, and an isogenic derivative carrying the *ospC* gene driven by the constitutive *flaB* promoter on the shuttle vector pBSV2G, termed A3/*flaB*_p_::*ospC*, was used for the constitutive *ospC* expression experiments [34, 35]. *B. burgdorferi* liquid cultures were propagated from frozen stocks in Barbour-Stoenner-Kelly II (BSK II) medium containing gelatin and 6 % rabbit serum, and supplemented with 40 μg/ml gentamicin, where appropriate. Viable spirochetes were quantified as colony forming units (CFUs) in solid BSK medium incubated at 35 °C with 2.5 % CO_2_, as described previously [36].

### Tick infection and infectious dose in mice

Mouse infection studies utilized 6-to-8 week old female mice of an outbred derivative of Swiss-Webster mice (termed RML) reared at the Rocky Mountain Laboratories breeding facility. Larval ticks acquired spirochetes by feeding on mice infected with WT spirochetes, as described previously [37]. Experiments to determine the 50 % infectious dose (ID_50_) in mice of WT spirochetes derived from unfed and fed infected nymphal ticks were conducted as previously described with minor modifications [37], and calculated by the method of Reed and Muench [38]. Briefly, two groups of 10 nymphs, before or 7 days after feeding on a naïve mouse, were surface sterilized by immersion first in 3 % hydrogen peroxide and then in 70 % ethanol, briefly air dried and subsequently ground in 1 ml BSK medium in a sterile 1.5 ml microfuge tube with a disposable plastic pestle (Kimble Chase, Rockwood, TN). This experiment was performed a second time using 30 unfed nymphs. For each group of fed or unfed nymphs, aliquots of undiluted tick homogenates were inoculated intradermally into mice using 100 μl per mouse. The remaining tick homogenates were serially diluted (range of 10^-1^ to 10^-5^), and 100 μl of each dilution was inoculated intradermally into mice. Aliquots of tick homogenates were also plated to enumerate CFU in the inocula. *B. burgdorferi* infection in mice was assessed by sero-conversion 3 weeks and isolation of spirochetes from cultures of ear, bladder and joint tissues 5 weeks after inoculation.

A second group of larval ticks was artificially infected by immersion [39] in cultures of WT or A3/*flaB*_p_::*ospC B. burgdorferi*; this route of infection was utilized because spirochetes that constitutively produce OspC are either cleared by the adaptive immune response of mice or persist as variants that no longer make OspC [40]. Following the molt, groups of 5-10 nymphs were ground in medium before or immediately after feeding to repletion on naïve mice, and the homogenates were aliquoted and frozen at -80 °C. At the start of animal experiments, aliquots were thawed and inocula targeting an approximate low (10^2^) and high (10^4^) spirochete dose per animal were injected into groups of mice. An aliquot of the unfed tick homogenate was also cultivated in medium for four days at 35 °C prior to inoculation; spirochete density expanded from ~10^5^cells/ml to 8 x 10^7^ cells/ml during this incubation period. Throughout this study, diluted aliquots of tick homogenates were plated before freezing and at the time of animal inoculation to determine the CFUs in homogenates and inocula. There was no significant loss of viability following freeze/thawing of tick homogenates. Individual unfed and fed ticks from these same cohorts were also homogenized and aliquots plated to determine spirochete burden per tick. *B. burgdorferi* infection in mice was assessed by sero-conversion 3 weeks and spirochete isolation from tissues 5 weeks after inoculation.

#### Ethical approval

Animal experiments were conducted following guidelines from the National Institutes of Health with protocols approved by the Rocky Mountain Laboratories Animal Care and Use Committee. The Rocky Mountain Laboratories are accredited by the International Association for Assessment and Accreditation of Laboratory Animal Care (AAALAC).

### Seroreactivity

Whole cell lysates of *B. burgdorferi* cultivated to late exponential phase (~7 x 10^7^ spirochetes/ml) were separated by polyacrylamide gel electrophoresis and transferred to nitrocellulose membranes following standard protocols [34]. A slot blot apparatus (Bio-Rad, Hercules, CA) was used to assess reactivity of individual mouse sera with *B. burgdorferi* proteins at 1:200 dilution, as described previously [37].

### Immunofluorescence assay (IFA)

The synthesis of OspC by WT or A3/*flaB*_p_::*ospC* spirochetes in unfed nymphal tick midguts was investigated by IFA and epifluorescence microscopy. Midgut tissues, dissected from unfed nymphs artificially infected as larvae [39] with WT or A3/*flaB*_p_::*ospC* spirochetes as described above, were fixed and incubated with a mixture of rabbit anti-*B. burgdorferi* polyclonal serum (provided by Tom Schwan [41]) and anti-OspC monoclonal antibody (provided by Robert Gilmore, [42]). Total *Borrelia* present and spirochetes synthesizing OspC were detected with a mixture of secondary antibodies containing rhodamine-conjugated goat anti-rabbit and FITC-labeled sheep anti-mouse, respectively.

### Statistical analysis

**Fig. 1:**
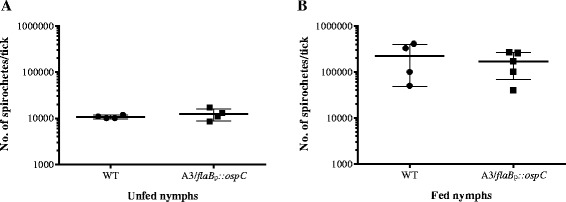
Spirochete loads in unfed and fed nymphs infected with WT and A3/*flaB*_p_::*ospC*. Nymphs artificially infected as larvae with WT *B. burgdorferi* or spirochetes engineered to constitutively produce OspC (A3/*flaB*_p_::*ospC*), were homogenized and plated **a** before and **b** after feeding on naïve mice to determine spirochete loads in ticks. The mean number of spirochetes (± SD) was similar between groups (*P* > 0.05, Student’s t-test, unpaired, 2-tailed) and increased approximately 10-fold when nymphs fed

The statistical significance of differences between spirochete loads in ticks (Fig. 1) was assessed using the GraphPad PRISM software with two-tailed, unpaired Student’s t-test with 95 % confidence interval (*P* < 0.05). The statistical significance of differences between mouse infections with unfed and fed tick homogenates (Tables 1 and 2) was assessed with pooled data using GraphPad software to compute two-tailed *P* values from Fisher’s exact test.

**Table 1:** Infectious dose in mice of WT*B. burgdorferi* derived from unfed and fed ticks

Spirochete source		Mouse infection	
	Inoculum^a^ (spirochetes/mouse)	Sero-positive mice^b^ (infected/no. inoculated)	Tissue isolation^c^ (infected/no. inoculated)
UNFED Ticks^d^			
Nymphs (group 1)	~1	0/5	0/5
	8	0/5	0/5
	80	0/5	0/5
Nymphs (group 2)	~10	0/5	0/5
	110	0/5	0/5
	1,100	0/5	0/5
	11,000	1/5	1/5
Adults	~1	0/5	0/5
	13	0/5	0/5
	130	0/5	0/5
	1,300	0/5	0/5
FED Ticks^e^			
Nymphs	~9	0/2	0/2
	90	5/5	5/5
	900	5/5	5/5
	9,000	5/5	5/5
	90,000	5/5	5/5

^a^No. of *B. burgdorferi* estimated by plating an aliquot of inocula for CFU

^b^Mice were bled 3 weeks after inoculation and assessed for seroconversion to *B. burgdorferi* whole cell lysates by immunoblot analysis

^c^Mice were euthanized 5 - 6 weeks after inoculation and infection determined by isolation of spirochetes from the ear, joint, and bladder tissues

^d^Groups of unfed nymphs were homogenized in medium and serially diluted for inoculation into mice (nymph groups (1) and (2) represent pools of 10 and 30 ticks, respectively)

^e^A group of 10 nymphs fed to repletion was homogenized in medium 7 days after drop-off and serially diluted for inoculation into mice

**Table 2:** Infectivity of tick-derived spirochetes engineered to constitutively produce OspC

Spirochete source and strain		Mouse infection	
	Inoculum^a^ (spirochetes/mouse)	Sero-conversion^b^ (infected/no. inoculated)	Tissue isolation^c^ (infected/no. inoculated)
UNFED Ticks			
WT	~90	0/5	0/5
	8,900	0/5	0/5
A3/*flaB*_p_::*ospC* ^d^	~70	0/5	0/5
	7,300	0/5	0/5
After Cultivation^e^			
WT	~10	0/5	0/5
	1,000	5/5	5/5
FED Ticks			
WT	~190	5/5	5/5
	18,800	5/5	5/5
A3/*flaB*_p_::*ospC*	~90	5/5	5/5
	8,800	5/5	5/5

^a^Groups of 5-10 infected ticks were ground in medium before or immediately after feeding to repletion on naïve mice, and homogenates frozen at -80 °C. Homogenates were thawed for inoculation of mice, and aliquots plated to determine the number of viable spirochetes in each inoculum

^b^Mice were bled 3 weeks post-challenge and sero-conversion to *B. burgdorferi* whole cell lysates assessed by immunoblot analysis

^c^Mice were euthanized 5 – 6 weeks post-challenge and infection assessed by attempted isolation of spirochetes from the ear, joint and bladder tissues of each mouse

^d^A3/*flaB*_p_::*ospC* refers to WT spirochetes engineered to constitutively produce OspC [15]

^e^Spirochetes in unfed tick homogenates previously frozen at -80 °C were thawed and cultivated in BSK II medium for 4 days at 35 °C prior to mouse inoculation. The number of viable bacteria injected was determined by plating an aliquot of each inoculum

## Results

### The ID_50_ in mice varies with *B. burgdorferi* source

Our previous studies calculated an ID_50_ in mice of ~500 in vitro-cultivated *B. burgdorferi*, while that of bacteria derived from nymphs fed for 72 h was ~10 spirochetes [37]. However, we had not determined the ID_50_ of spirochetes obtained from nymphs prior to feeding (unfed ticks). Other investigators had demonstrated that homogenates derived from unfed nymphs were not infectious for hamsters or mice, and concluded that spirochete replication during tick feeding was required to attain an infectious dose [20, 21, 23]. To determine the approximate number of spirochetes from unfed nymphs required to infect a mouse, larval ticks infected by feeding on WT-infected mice were allowed to molt to nymphs, and nymphal ticks were sampled before and after feeding to repletion on naïve mice. Using a dilution series of homogenates prepared from unfed and fed ticks, we challenged mice by needle inoculation with tick-derived *B. burgdorferi* and assessed infection. Only 1/45 mice became infected following injection of unfed tick homogenates containing from ~10 to 10,000 spirochetes, whereas 17/20 mice were infected with a comparable range of inocula from fed ticks (*P* < 0.0001) (Table 1). This outcome does not permit an infectious dose estimate for spirochetes in unfed ticks (although it must be greater than 10^4^), but yields an ID_50_ of ~30 organisms for *B. burgdorferi* derived from replete nymphs 7 days after drop off, similar to previous determinations [37]. These data establish that viable spirochetes in unfed ticks are highly attenuated relative to spirochetes obtained from recently fed ticks.

### Constitutive production of OspC by spirochetes in unfed ticks does not restore virulence

**Fig. 2:**
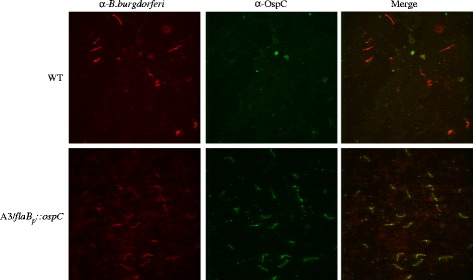
OspC production by WT and A3/*flaB*_p_::*ospC* spirochetes in unfed nymphs. Nymphs were artificially infected as larvae with wild-type *B. burgdorferi* strain B31-A3 (WT) or an isogenic derivative engineered to constitutively express *ospC (*A3/*flaB*_p_::*ospC*). Spirochetes in dissected tick midguts were detected by IFA with a polyclonal anti-*B. burgdorferi* primary antiserum [41] and rhodamine-labeled secondary antibody, while synthesis of OspC was examined using a monoclonal anti-OspC primary antibody [42] and FITC-labeled secondary antibody

The production of OspC, an essential virulence factor for mammalian infection [14], ceases during tick acquisition of *B. burgdorferi* from an infected mammal and the protein remains undetectable on spirochetes until the subsequent tick blood meal [13, 41]. Because spirochetes derived from unfed ticks were essentially avirulent in mice (Table 1), we wished to determine whether engineered constitutive production of OspC could restore infectivity. To do this, larval ticks were infected by immersion [39] in cultures of WT spirochetes or an isogenic derivative engineered to constitutively produce OspC (A3/*flaB*_p_::*ospC*) [35], and then fed to repletion on naïve mice. Larval ticks were allowed to molt and spirochete loads were determined for both cohorts before and after the nymphal bloodmeal. There was a 10-fold increase in spirochete loads in nymphs after feeding, and similar numbers of bacteria were detected for both strains (*P* > 0.05; Fig. 1), indicating that there was no difference between strains in their ability to colonize, persist and replicate in nymphs. We also assessed OspC production by spirochetes in unfed nymphal tick midguts using immunofluorescence. As expected, none of the WT spirochetes were OspC-positive, whereas all A3/*flaB*_p_::*ospC* spirochetes present in an unfed nymph made OspC (Fig. 2).

Next, we assessed the infectivity in mice of both groups of tick-derived spirochetes. Using homogenates from unfed or fed infected nymphs, naïve mice were inoculated with approximately 10^2^ and 10^4^ spirochetes. None of the mice (0/20) inoculated with WT or A3/*flaB*_p_::*ospC* from unfed tick homogenates became infected, whereas all mice (20/20) challenged with fed tick homogenates carrying either strain became infected and spirochetes were isolated from all tissues tested (*P* < 0.0001) (Table 2). We did not assess shuttle vector retention in isolates from mice inoculated with A3/*flaB*_p_::*ospC*, but we would expect the constitutively expressed *ospC* gene to be lost in the majority of spirochetes by 5 weeks of infection, as previously demonstrated to be necessary for persistence of these spirochetes after immune recognition of OspC [35, 40, 43]. Additionally, WT organisms that grew out from liquid cultivation of unfed tick homogenates were infectious in mice (5/5) with an inoculum of ~10^3^ spirochetes (Table 2), consistent with previous estimates of infectivity with in vitro grown organisms [37]. Importantly, these data demonstrate that tick feeding primes *B. burgdorferi* for infection in mice by induction of critical components in addition to the known virulence factor OspC.

## Discussion

The Lyme disease spirochete must undergo specific physiological changes in order to adjust and survive in the different environments encountered during its natural infectious cycle (reviewed in [5, 6]). Spirochetes must also migrate from the tick midgut to the salivary glands in order to be transmitted, a process that typically takes ~48 h [23, 26, 44–47]. We designed the present study to quantitatively assess the virulence of spirochetes derived from ticks before and after feeding on a vertebrate host. We needle-inoculated mice with infected tick material to bypass requirements specific for tick transmission and focus the comparison on the relative capabilities of these spirochetes to infect a mammalian host. Viable spirochetes in these inocula were quantified by colony formation in solid medium. A significant finding of this study was that 0/10 mice were infected with an inoculum of ~10^3^ viable spirochetes from unfed ticks and only 1/15 mice became infected with inocula ranging from ~7×10^3^ to 1×10^4^ organisms from this source (Table 1). This outcome does not permit calculation of the ID_50_ for spirochetes from unfed ticks, whereas we calculated an ID_50_ of ~30 for spirochetes from fully engorged ticks. Increasing the inoculum 10-fold to 10^5^ organisms in order to potentially determine an ID_50_ for spirochetes in unfed ticks would require injecting the undiluted homogenate of ~ 30 unfed ticks per mouse (Table 1), which is technically limiting. When the outcomes of separate experiments are considered together (Tables 1 and 2), only 1/65 mice became infected following inoculation of ~ 10 to 10^4^ spirochetes from unfed ticks, whereas 35/37 mice were infected by similar numbers of tick-derived spirochetes after the bloodmeal. Together these results demonstrate a large increase in the virulence of spirochetes when they have been “primed” by a blood meal. Previous studies have reported the lack of infectivity of *B. burgdorferi* from unfed ticks, but this outcome was attributed to potentially low numbers of bacteria in the inocula, which were difficult to accurately measure [20, 23]. However, our findings indicate that in addition to physical location and absolute number, the virulence of resident spirochetes is fundamentally different after tick feeding commences.

One well-characterized and major difference between *B. burgdorferi* in unfed versus fed ticks is the differential production of outer surface proteins, with OspA produced by spirochetes in unfed ticks and OspC induced during tick feeding [13, 41, 48]. The presence of OspA on spirochetes has been correlated with successful colonization of the tick midgut [49–51], while induction of OspC during tick feeding is an absolute requirement for *B. burgdorferi* to initiate infection of the vertebrate host [14, 15]. Therefore, since *B. burgdorferi* in unfed ticks do not produce OspC, it is perhaps not surprising that spirochetes persisting within an unfed tick environment would be unable to infect mice. However, we demonstrated that spirochetes derived from unfed ticks failed to infect mice even when they were engineered to constitutively produce OspC (Table 2). These data indicate that the presence of the virulence factor OspC is not sufficient to ‘prime’ spirochetes coming out of unfed ticks for productive mouse infection, and further demonstrate that induction of OspC is only one of several critical adaptive responses that spirochetes undergo during tick feeding to prepare for host infection [31, 32, 52].

There are plausible explanations for the observed lack or significant attenuation of virulence of spirochetes in the unfed tick vector other than OspC. The RpoN-RpoS regulatory cascade, which governs expression of many *B. burgdorferi* genes in the mammalian host, is shut off during spirochete acquisition by feeding ticks and remains off until it is activated during the next blood meal [11, 12, 32, 52]. A recent study by Iyer and colleagues utilized an amplification-microarray approach to compare the transcriptomes of mammalian host-adapted spirochetes with those in fed ticks or cultivated in vitro [31]. Significant differences were noted in the global patterns of gene expression among spirochetes from these distinct sources, particularly in various aspects of metabolism, nutrient uptake and chemotactic response [31]. These spirochetes were all in metabolically active states supported by nutrients present in the host, the fed tick or culture medium, whereas metabolically inactive spirochetes in unfed nymphal ticks (which we found to be non-infectious) were not part of this comparison because they do not provide enough material for microarray analysis. A direct comparison of global gene expression between spirochetes from fed and unfed ticks would be extremely insightful, however, and the substantially larger unfed adult tick, whose spirochete burden is similarly non-infectious (Table 1), but approximately 50-fold higher than that of an unfed nymph (data not shown), could represent a good source of material for such future analyses.

Genes with RpoS-dependent expression patterns like *ospC* (abundantly transcribed by spirochetes in the host and in fed nymphal ticks, but expressed at very low levels by spirochetes in fed larval ticks), should provide insight into virulence factors specifically induced for host infection rather than stimulated for cell growth by the bloodmeal [31, 32]. Surprisingly, of the 100 genes most abundantly expressed by spirochetes in fed nymphs, only *ospC* exhibited this anticipated pattern of putative virulence factor expression [31]. In addition, only a few members of the previously identified set of RpoS-dependent genes of *B. burgdorferi* [11] appear in the “top 100” list in fed nymphs, and of these, *ospC* is the only gene that is also highly expressed by host-adapted spirochetes [31]. It seems unlikely that metabolic state and OspC production, while both critically important, are the sole determinants of the infectious phenotype of spirochetes in fed versus unfed nymphs. Rather, less abundant gene products not highlighted by microarray analyses are also likely to play a key role in preparing *B. burgdorferi* for mammalian infection. Likewise, transcriptome comparisons do not identify post-transcriptional regulatory mechanisms that alter translation or protein turnover, which have been shown to play a role in modulation of RpoS function [53, 54]. Some spirochetal components, such as the integral outer membrane protein P66, are induced during tick feeding independently of the RpoN-RpoS regulon, yet are essential in the host [55, 56]. Finally, other *B. burgdorferi* factors that are specifically made during the tick starvation period could actively impede spirochete infectivity in the mammalian host [16, 24, 25, 27, 32, 53, 57, 58]. Thus, both the presence and absence of particular factors could contribute to the avirulent phenotype of spirochetes in unfed ticks.

## Conclusions

Our current study illustrates the impact of the vector environment on the physiological or biological state of the Lyme disease spirochete, which in turn directly influences its virulence in the mammalian host. Spirochete replication as a consequence of the blood meal is clearly important during the natural route of transmission by tick bite. However, our findings demonstrate that a fundamental difference between spirochetes in unfed and fed ticks is their exposure and subsequent response to a key priming event that enables mammalian infection. This study and others (for example [13, 27, 31, 32, 48]) underscore the need for direct analysis of *B. burgdorferi* within the tick vector throughout the transmission cycle. Such investigations will provide a better understanding of the basic biology of the Lyme disease spirochete, which forms a basis for rational design of preventive and therapeutic interventions for human infection.

## Abbreviations

WT: wild type
CFU: colony forming unit
Osp: outer surface protein
IFA: immunofluorescence assay
ID_50_: dose needed to infect 50 % of challenged recipients
